# Facile synthesis of CsPbBr_3_ perovskite for improved stability and luminescence behavior in an aquatic environment

**DOI:** 10.3389/fchem.2025.1524254

**Published:** 2025-02-25

**Authors:** Xianqi Wei, Jiayi Lu, Silong Zhang, Jiashu Tang, Shichao Wu, Tengbo Lv, Xiaoli Wang

**Affiliations:** ^1^ School of Science, Jiangsu Ocean University, Lianyungang, Jiangsu, China; ^2^ School of Microelectronics, Xi’an Jiaotong University, Xi’an, Shaanxi, China; ^3^ School of science, Xi’an Jiaotong University, Xi’an, Shaanxi, China

**Keywords:** CsPbBr3 perovskite, strontium, aqueous solutions, stability, luminescence

## Abstract

CsPbBr_3_ (CPB) perovskite has demonstrated unique advantages as a photoelectric material. However, its stability and optoelectronic properties exhibit significantly susceptibility to environmental conditions during practical applications. Additionally, the synthesis of CPB often involves complex procedures and stringent requirements for the experimental environment, resulting in low yield. In this study, we employed an aqueous-phase synthesis method to incorporate strontium into CPB, aiming to enhance the long-term stability of the perovskite in aqueous solutions. And the introduction of strontium (Sr) is expected to improve the photoluminescent properties of the perovskite. The results demonstrate that the synthesized perovskite remains stable in aqueous solution for up to 264 h, with enhanced photoluminescence intensity and a blue shift attributed to the incorporation of strontium. This approach significantly increases the potential value of CPB perovskite for applications in optoelectronic materials and devices.

## 1 Introduction

CsPbBr_3_ (CPB) has attracted significant attention in recent years as a promising perovskite material with exceptional optoelectronic properties. Its unique characteristics present remarkable application potential in cutting-edge fields, including the development of advanced optoelectronic materials, the design of efficient solar cells, the exploration of innovative photocatalytic technologies, and the construction of highly sensitive photodetection systems. In-depth investigations into the properties and functionalities of CPB perovskite could catalyze technological advancements in these domains and facilitate a series of innovative breakthroughs (Chen et al., 2022; Duong et al., 2022). Such developments are crucial for addressing pressing global challenges, including the energy crisis, environmental pollution monitoring, and advancements in information technology. The pursuit of these applications presents both challenges and opportunities for enhancing the optoelectronic performance of CPB (Swarnkar et al., 2015; Wu et al., 2021; Son et al., 2023).

Nevertheless, the environmental sensitivity of CPB, particularly with respect to humidity, temperature, and light exposure, plays a crucial role in determining its stability and optoelectronic performance. For instance, CPB exhibits a pronounced susceptibility to degradation in humid conditions, leading to a marked decline in its optoelectronic properties. Moreover, under intense light exposure, CPB may undergo photo-degradation, compromising stability over prolonged use and ultimately diminishing the efficiency and lifespan of devices in practical applications (Vighnesh et al., 2022). In high-temperature conditions, the degradation rate of CPB accelerates, and its crystal structure may undergo rapidly changes, resulting in a sharp decline in stability and significantly impacting its optical and electrical properties. Additionally, the relatively high charge recombination rate of CPB during practical applications significantly limits its photocurrent conversion efficiency, thereby constraining its development prospects (Chen et al., 2018). Previous preparation methods for CPB have also encountered unavoidable defects, such as complex operational procedures, stringent experimental conditions, and low synthesis yields, all of which increase the difficulty and cost of experiments (Wei et al., 2020; Yin et al., 2021; Zhou et al., 2021).

To address these issues, we have adopted an aqueous phase method to synthesize CPB perovskite, incorporating rare earth strontium elements during the synthesis process. This approach aims to enhance the stability of CPB in aqueous environments and modulate its luminescent properties due to the incorporation of strontium. By employing this method, we hope to overcome the deficiencies of traditional synthesis techniques, optimize the preparation process, improve long-term water stability in aqueous solution, and increase synthesis efficiency, thereby providing a more stable and effective solution for the practical applications of CPB. Additionally, the aqueous synthesis method is anticipated to lower costs and simplify operations, paving the way for new avenues in the research and application of perovskite materials.

## 2 Methods

### 2.1 Materials

N-N-dimethylacetamide (DMA, 99.9%), Lead bromide (PbBr2, 99.999%), Cesium bromide (CsBr, 99.99%), Cesium trifluoroacetate (Cs-TFA, 98%), 4-bromo-butyric acid (BBA, 98%), Oleylamine (OLA, 80%–90%), and Strontium Carbonate (SrCO_3_, ≥99.95%) were purchased from Aladdin.

### 2.2 Experimental design

Herein, we prepared CPB perovskite containing rare earth Sr elements (CPB/Sr) by strontium carbonate (SrCO_3_) using a aqueous-based method in an aqueous solution. The entire synthesis process of CPB/Sr was conducted in a aqueous solution environment. This method yields CPB/Sr perovskite with enhanced water stability compared to those synthesized via other methods, which tend to decompose easily upon contact with water or even degrade in humid environments, making it difficult for perovskite to exist in aqueous conditions.

**FIGURE 1:**
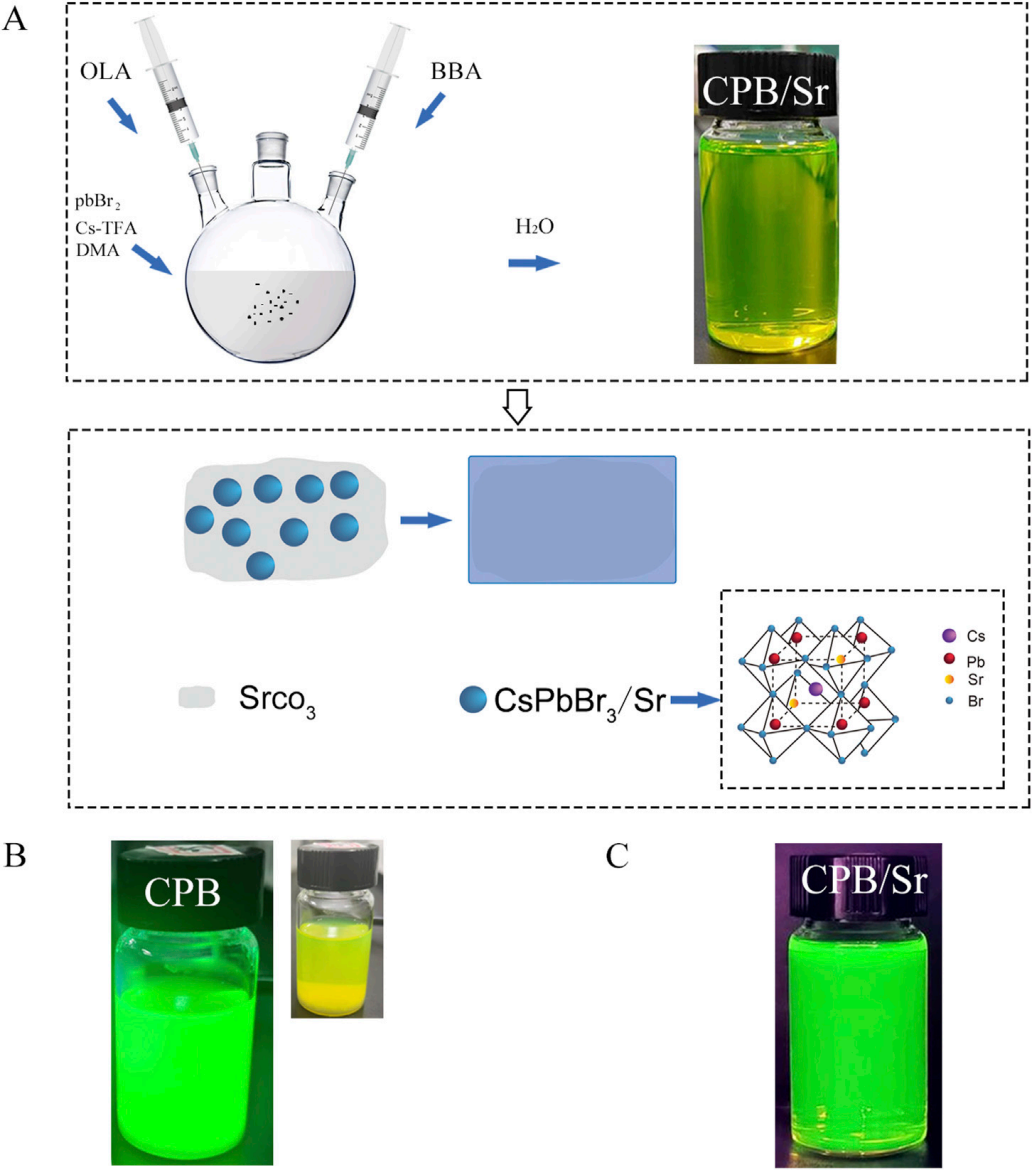
CsPbBr3 (CPB) perovskite with strontium (Sr) by strontium carbonate (SrCO3) using a aqueous-based method in aqueous environment. **(A)** synthesis mechanism of CPB with Sr. **(B)** the image of CPB Perovskite in Aqueous Solution under 365 nm UV light illumination and natural light; **(C)** the image of CPB/Sr under 365 nm UV light illumination.

The synthesis procedure by adding 146.8 mg of SrCO_3_ powder to 10 mL of N,N-Dimethylacetamide (DMA) solvent and subjected the mixture to ultrasonic stirring for 4 h to ensure uniform dispersion of SrCO_3_ particles in DMA solvent for the synthesis of CsPbBr_3_. The 10 mL DMA solution was then transferred to a three-neck flask, where 146.8 mg of lead bromide (PbBr_2_) and 146.8 mg of cesium trifluoroacetate (Cs-TFA) were added. In this process, PbBr2 provides the lead ions required for the perovskite structure, while Cs-TFA serves as the cesium source. The temperature of the magnetic stirrer was then raised to 60°C for 45 min of stirring. Subsequently, 0.2 mL of oleylamine (OLA) and 0.65 mL of 4-bromobutyric acid (BBA) were added to the solution. OLA aids in controlling the size and morphology of the quantum dots, while BBA provides a bromine-rich environment and carboxylate groups for the synthesis of CPB. The mixture was stirred for an additional 15 min to ensure thorough mixing, resulting in a precursor solution. Finally, 2 mL of the precursor solution was injected into 25 mL of deionized water to synthesize CPB/Sr perovskite quantum dots in an aqueous environment, as shown in Figure 1. Figure 1A shows formation mechanism of CPB/Sr. Figure 1B visibly indicates that the CPB/Sr perovskite is clear and transparent with a yellow-green, without any aggregation or precipitation. Figure 1C, under 365 nm UV light, also shows the solution to be clear and transparent with a fluorescent green color, exhibiting no turbidity or aggregation.

This method offers a simple and easy-to-operate process for preparing the precursor solution of CPB/Sr. The precursor is directly used to synthesize CPB/Sr in a single step within an aqueous medium. This approach overcomes the shortcomings of traditional synthesis methods, optimizes the preparation process, and enhances synthesis efficiency, providing a viable pathway for the large-scale market application of perovskite.

### 2.3 Instrument

The surface morphology of CPB/Sr perovskite was investigated by transmission electron microscope (TEM) and high-resolution TEM (G2F30, FEI Tecnai, America). The X-ray diffraction (XRD) patterns were carried out by the nickel filtered Cu Kα radiation in the 2θ range 10°–60° at a scan speed of 1.0° 2θ min^-1^ (Ultima IV, Rigaku, Japan). Fourier transform infrared spectroscopy (FTIR) was studied by an FTIR spectrometer (Nicolet IS-10, Thermo Fisher Scientific, America). The X-ray photoelectron spectroscopy (XPS) was obtained by an X-ray electron spectrometer (ESCALAB Xi, Thermo Scientific, America). The Ultraviolet-Visible (UV-Vis) absorption spectra were investigated by a UV-Vis spectrophotometer (UV-1800PC, Shanghai MEPULDA Instrument Co., China). The photoluminescence (PL) spectra was measured by a fluorescence spectrophotometer (WFY-28, Tianjin Topu Instrument Co., China) applying a 405 nm laser of 0.5 W as the excitation source. Time resolved PL decay curves were carried out using a fluorescent lifetime spectrometer (FLS1000, Edinburgh, Britain) employing the time-correlated single photon counting technique with excitation provided by a picosecond pulsed diode laser operating at a wavelength of 375 nm.

## 3 Result and discussion

**FIGURE 2:**
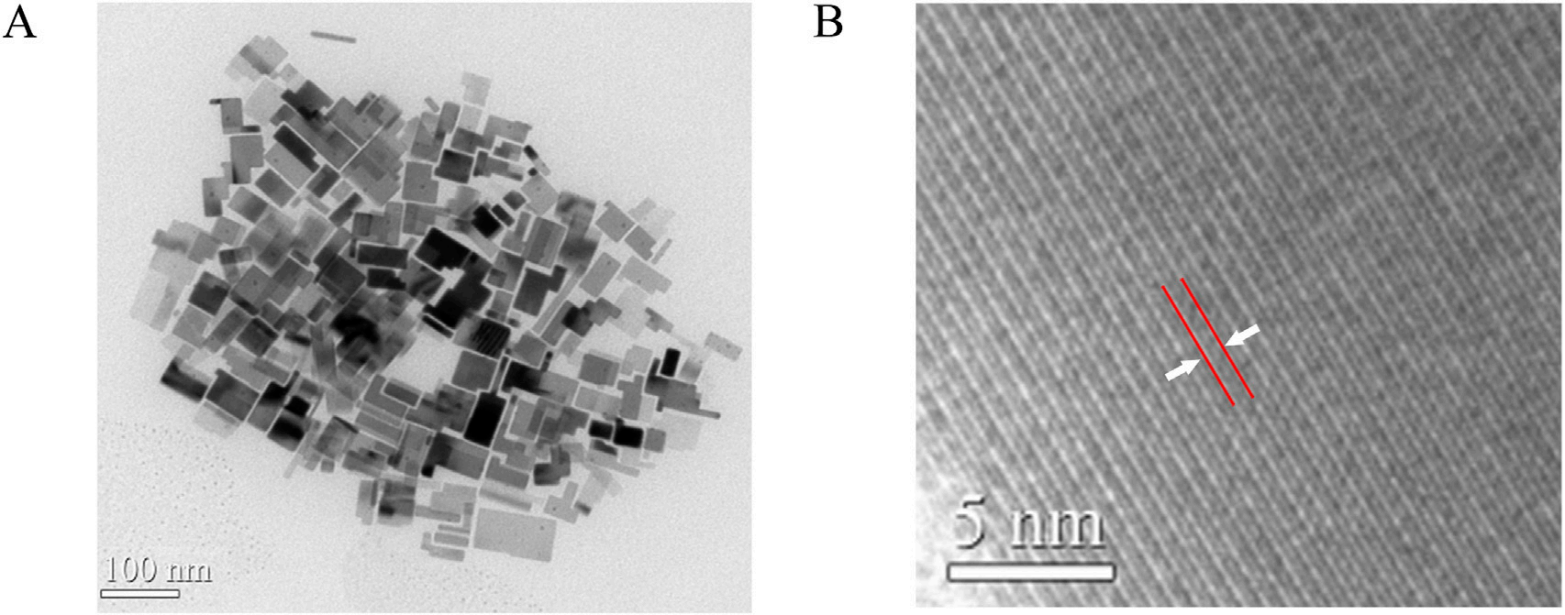
**(A)** TEM image of CPB perovskite with Sr element. **(B)** high-resolution TEM image showing lattice fringe with 0.59 nm.

**FIGURE 3:**
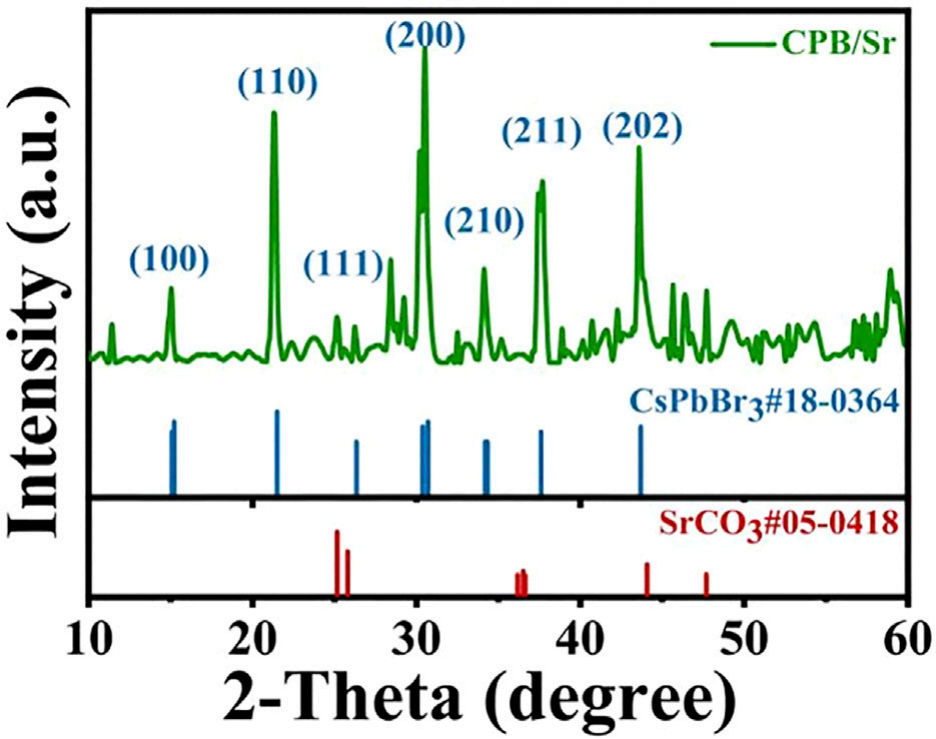
XRD patterns of CPB perovskite with Sr element.

We synthesized CPB/Sr perovskite using a one-step aqueous method and characterized its microstructure through transmission electron microscopy (TEM), as shown in Figure 2. From Figure 2A, the results indicate that the nanocrystals exhibit high crystallinity, distinct grain boundaries, and a block-like grain morphology. Figure 2B presents a high-resolution TEM image, where lattice fringes can be observed, with a lattice spacing of 0.59 nm, corresponding to the (100) crystal plane of cubic CPB. The microstructure of the CPB/Sr nanocrystals was further characterized using X-ray diffraction (XRD), as illustrated in Figure 3. A comparison with the standard PDF card #18–0364 for CsPbBr3 nanocrystals and PDF card #05–0418 for SrCO3 reveals successful synthesis of the CPB containing element Sr. The XRD spectrum shows several distinct characteristic peaks between 20° and 60°, with peaks at 15.2°, 21.3°, 25.2°, 30.5°, 34.1°, 37.7°, and 43.6° corresponding to the (100), (110), (111), (200), (210), (211), and (202) crystal planes of CsPbBr3, respectively. Additionally, the peaks at 25.2°, 37.7°, 44.1°, and 47.7° correspond to the (111), (211), (221), and (132) diffraction planes of SrCO3(Møller, 1958; Tuan Minh et al., 2022; Wan et al., 2024). These results confirm that we have synthesized a cubic phase structure of CPB perovskite, which grows on the surface of SrCO_3_.

**FIGURE 4:**
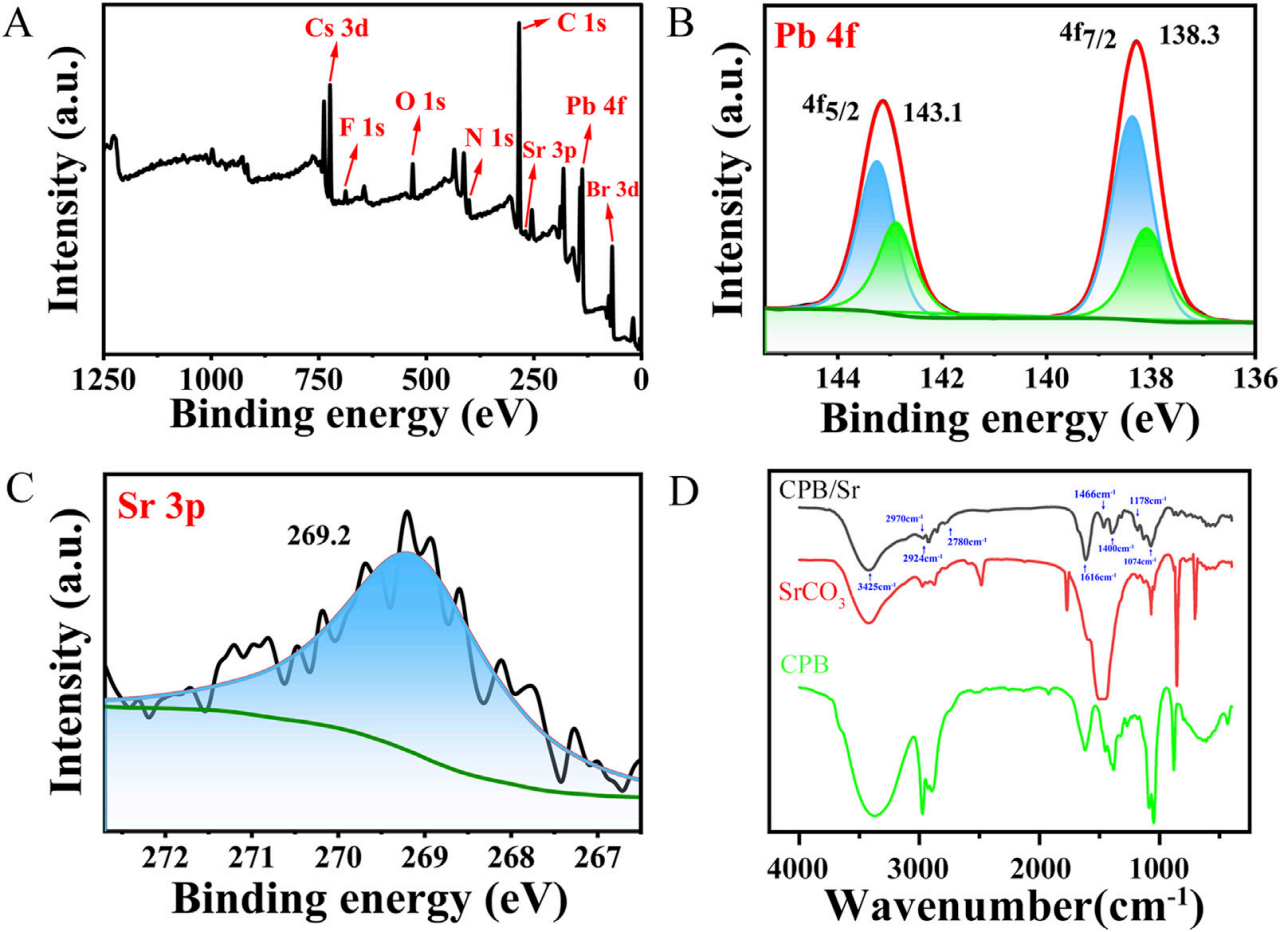
**(A)** XPS full energy spectrum of CPB/Sr. **(B)** XPS spectrum of Pb 4f. **(C)** XPS spectrum of Sr 3p. **(D)** FTIR spectra of CPB perovskite with Sr.

To gain a deeper understanding of the chemical bonding and surface interactions between the Sr element and CPB perovskite, we performed an X-ray photoelectron spectroscopy (XPS) analysis on the elements Cs, Pb, Br, and Sr, illustrating in Figure 4. As shown in Figure 4A, a distinct F 1s peak appears at 688.2 eV, which is attributed to the presence of TFA ligand ions that bind to the surface of the CPB nanocrystals. The N 1s peak is associated with the ligand OLA. Oxygen (O) is present not only related to Cs-TFA, BBA, OLA, SrCO_3_ ligands, but also to the fact that the experimental process was not conducted under vacuum conditions. The characteristic Br 3 days peak originates from the BBA ligand during the crystal synthesis process. The Sr 3P spectrum exhibits a main peak at 269.2 eV, which is attributed to the introduction of SrCO_3_ (Jisong et al., 2024). The Pb 4f spectrum displays characteristic peaks at 138.1 eV (Pb 4f_7/2_) and 142.9 eV (Pb 4f_5/2_), while two corresponding main peaks of Pb 4f measured at 138.3 eV (Pb 4f_7/2_) and 143.1 eV (Pb 4f_5/2_) in Figure 4B, which shift to higher binding energy. Figure 4C shows XPS spectrum of Sr 3p. It is because when Sr element participates in the synthesis process of CsPbBr_3_ in the form of SrCO_3_ solvent, effectively passivates the uncoordinated Pb^2+^ on the nanocrystalline surface and reduces the charge density around Pb^2+^, leading to a contraction of the perovskite lattice structure and stronger binding energy, as reflected in the shift of the XPS peak positions (Jialong et al., 2018; Wenming et al., 2023).

To further analyze the chemical composition and bonding characteristics of CPB/Sr, Fourier-transform infrared (FTIR) spectroscopy was conducted on CPB/Sr, SrCO_3_, and BBA, as illustrated in Figure 4D. The broad peak at 3,425 cm⁻^1^ is attributed to the stretching vibrations of O-H bonds on the surface of CPB/Sr, resulting from its crystallization in water. Peaks at 2,970 cm⁻^1^ and 2,924 cm⁻^1^ correspond to the symmetric and asymmetric stretching vibrations of C-H bonds, associated with hydrocarbon chain interactions (Neda et al., 2019). The stretching vibration at 2,780 cm⁻^1^ and the bending vibration of O-H at 1,400 cm⁻^1^ arise from carboxylic acids in BBA. The absorption peak at 1,616 cm⁻^1^ represents the C=O stretching vibration, which is strong and sharp, indicating a shift from 1,685 cm⁻^1^–1,616 cm⁻^1^ upon the addition of BBA and Cs-TFA (Kunnathodi et al., 2022). Following the introduction of BBA and Cs-TFA, the carboxylate groups provided by BBA and TFA⁻ coordinate with Pb^2^+ to form Pb-O bonds. In this interaction, C=O acts as an electron donor, while Pb^2^+ acts as an acceptor, facilitating the formation of Pb-O bonds through coordination with O atoms provided by CF_3_CO_2_⁻ and BBA. The absorption peak at 1,466 cm⁻^1^ is due to the influence of CO_3_^2^⁻ groups from the addition of SrCO_3_, while the sharp peak at 1,074 cm⁻^1^ is attributed to the symmetric stretching vibrations of CO_3_^2^⁻ from SrCO_3_. The absorption peak at 1,178 cm⁻^1^ results from the stretching vibrations of C-F bonds in Cs-TFA. The absorption bands between 800 cm⁻^1^ and 500 cm⁻^1^ are associated with C-Br bond vibrations (Sami and Mohamed Ben, 2023; Li et al., 2024). These results suggest that O and Pb elements interact to form Pb-O bonds, eliminating Br vacancy defects. Additionally, due to its high electronegativity, F generates a strong electron effect when in proximity to C atoms. The formation of C-F bonds facilitates the construction of a hydrophobic shell, thereby enhancing the stability of the CPB perovskite nanocrystals and resisting their degradation.

**FIGURE 5:**
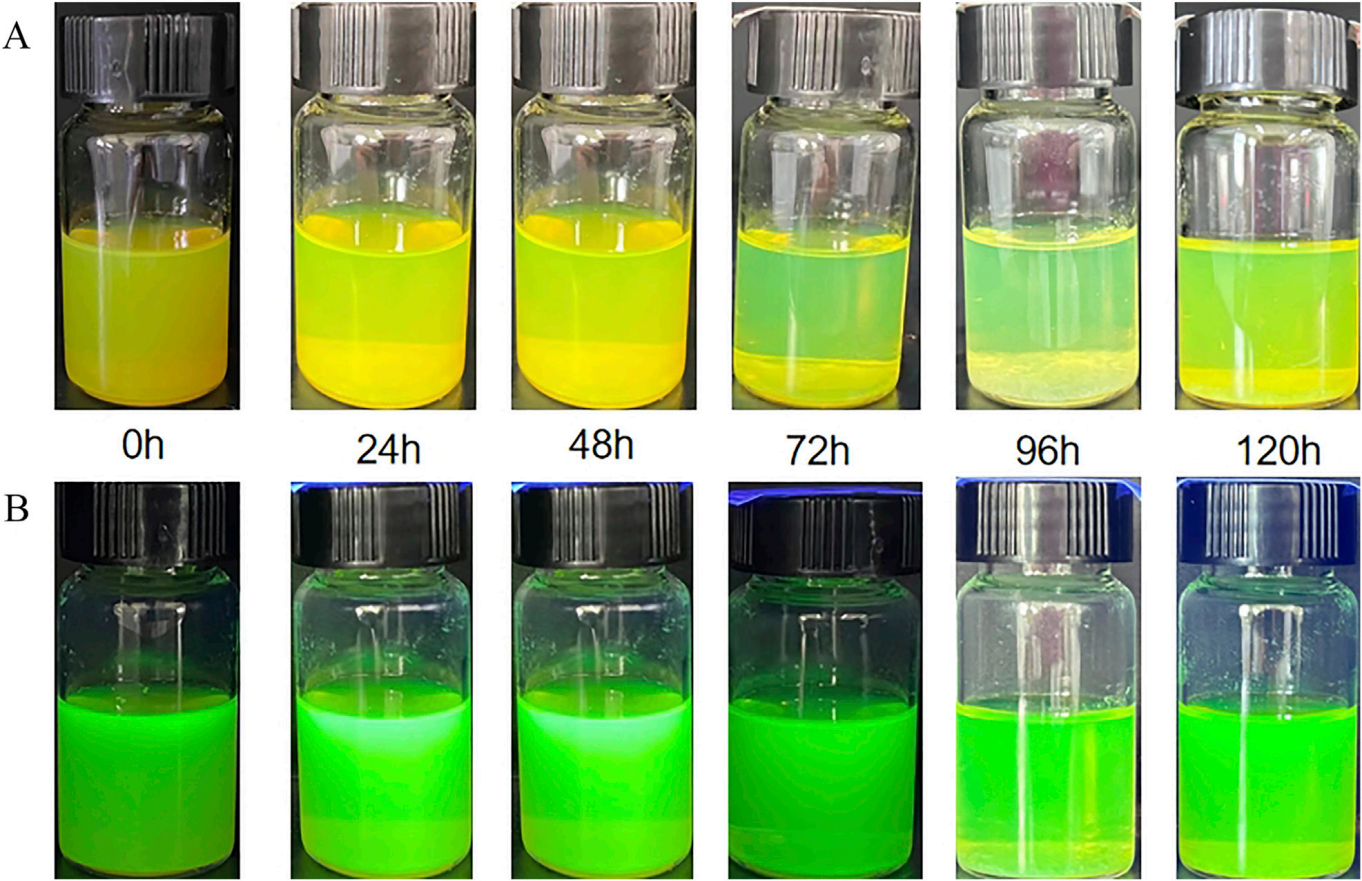
Presents sample images of CPB/Sr as a function of time. **(A)** shows the time-dependent changes of CPB/Sr perovskite under natural light; **(B)** depicts the time-dependent changes of CPB/Sr perovskite under 365 nm UV light.

Figure 5 shows the time-dependent changes of CPB/Sr perovskite nanocrystals in aqueous solution under both natural light and 365 nm UV light. From the figure, it can be observed that the prepared CPB/Sr perovskite nanocrystals exhibit no significant color change after continuous observation for 120 h in aqueous solution, under both natural light and 365 nm UV irradiation. Figure 5A displays a photograph of the synthesized CPB/Sr perovskite in aqueous solution at room temperature over a period of 120 h. Initially, the solution appeared yellow-green, gradually becoming lighter and more transparent over time. This change is attributed to the slow decomposition of the perovskite nanocrystals in water. At present, most of the prepared perovskites tend to decompose upon contact with water, however the nanocrystals synthesized via the aqueous method in this paper retained their integrity for 120 h without complete decomposition showing. This observation highlights their excellent stability in aqueous environments by the aqueous method. Figure 5B illustrates the fluorescence images of CPB/Sr perovskite nanocrystals in aqueous solution under 365 nm UV light, taken at various time points (0 h, 24 h, 48 h, 72 h, 96 h, and 120 h) at room temperature. It is evident from the images that the samples bright and distinct green fluorescence when exposed to 365 nm UV light, demonstrating their superior photoluminescent properties. Moreover, after 120 h of observation, the fluorescence intensity remained high, with no significant change in the emission characteristics. These results indicate that the CPB/Sr perovskite nanocrystals synthesized by the aqueous method exhibit not only structural stability but also exceptional luminescent properties over extended periods in aqueous environments.

**FIGURE 6:**
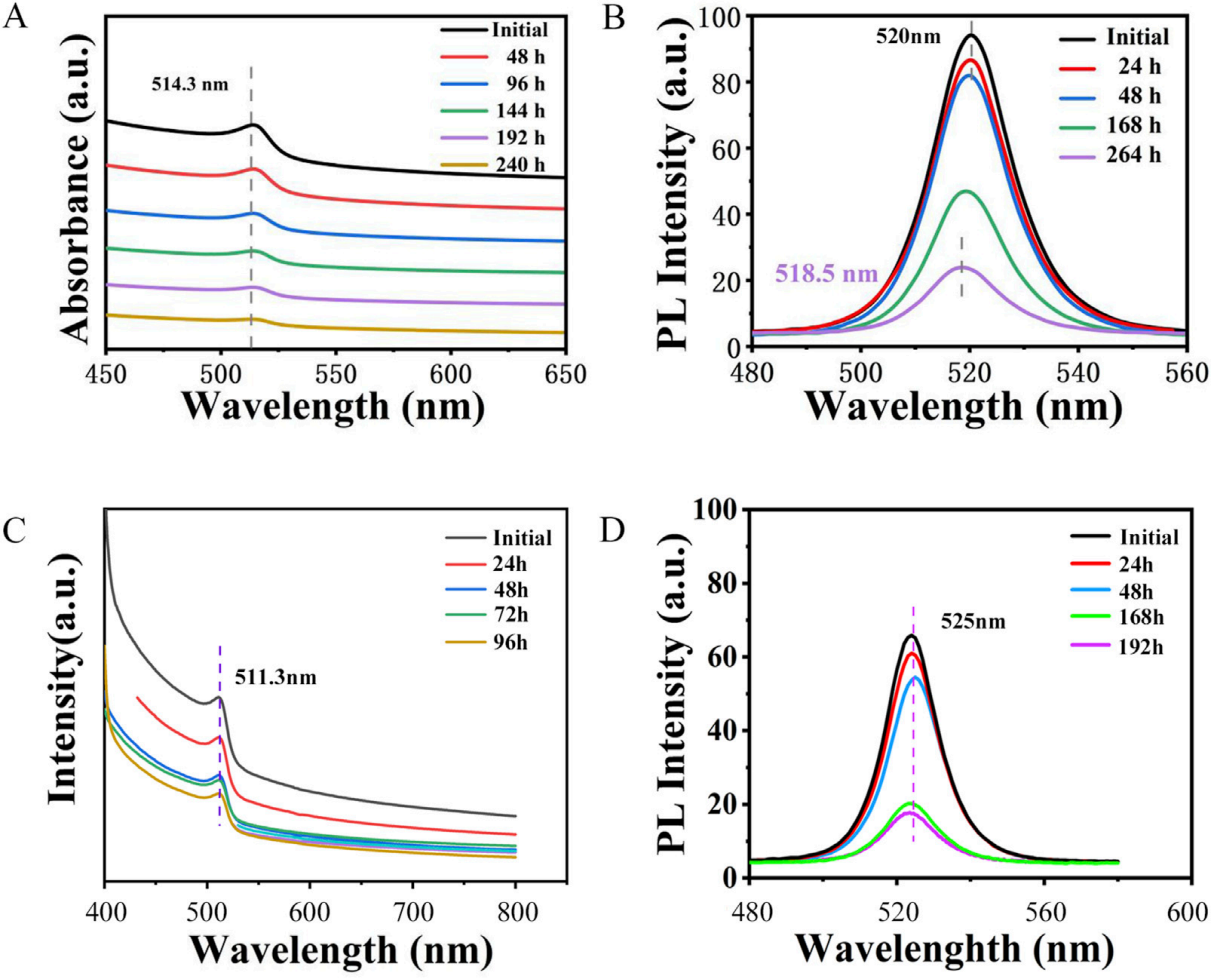
**(A)** UV-vis absorption spectra of CPB/Sr. **(B)** fluorescence emission spectra of CPB/Sr. **(C)** UV-vis absorption spectra of CPB. **(D)** fluorescence emission spectra of CPB.

To further investigate the luminescent properties of the synthesized CPB/Sr, we analyzed its absorption and photoluminescence (PL)spectra comparing with CPB synthesized by the same method, as shown in Figure 6. Figure 6A presents the absorption spectra of the CPB/Sr perovskite nanocrystals, recorded at 48-hour intervals over a total period of 240 h. A comparison of the absorption spectra of CPB/Sr at 0 h, 24 h, 96 h, 144 h, 192 h, and 240 h, within the 450 nm–650 nm wavelength range, shows that the central characteristic peak of CPB/Sr remains at 514.3 nm even after 240 h, with only minor changes in intensity. This stability suggests that the CPB/Sr perovskite undergoes minimal decomposition in water over time, leading to only a slight reduction in intensity. Therefore, the absorption spectrum observed after 240 h further confirms the structural integrity and excellent water stability of the CPB/Sr nanocrystals, in agreement with the findings presented in Figure 5. CPB absorption spectra shows in Figure 6C. Figure 6B presents the PL spectra of the prepared CPB/Sr perovskite nanocrystals in an aqueous environment at 0 h, 24 h, 48 h, 168 h, and 264 h, respectively. As observed from the figure, the central characteristic peak initially at 520 nm gradually shifts to the left to 518.5 nm after 264 h, indicating a slight blue shift in the central wavelength peak. This shift is attributed to changes in the bandgap of the sample due to the gradual decomposition and structural instability of the Sr-doped CPB perovskite nanocrystals over 264 h. However, within the first 48 h, the PL intensity remains largely unchanged, and even after 264 h, CPB still exhibits a distinct PL central peak. When compared to the PL spectrum of pure CPB in Figure 6D, the PL emission intensity of CPB is significantly weaker than that of CPB/Sr after 168 h, suggesting that CPB/Sr decomposes more slowly than CPB in an aqueous environment, thus exhibiting greater stability. Furthermore, the PL emission peak of CPB is located at 525 nm. Comparing this with the PL emission peak of CPB/Sr, it is evident that the PL emission peak of Sr-doped CPB perovskite undergoes a blue shift. This blue shift is likely due to the incorporation of Sr^2+^ from partially dissolved SrCO_3_ into the synthesis of the perovskite nanocrystals, potentially altering the band structure of the material, increasing the energy difference between the valence and conduction bands, and enhancing the energy of the emitted light, thereby optimizing the optical properties.

Time-resolved photoluminescence (TRPL) spectroscopy is a technique used to determine carrier lifetimes by measuring the variation of fluorescence intensity over time after excitation. This method can be employed to calculate the fluorescence lifetime of the material. The tri-exponential decay function is commonly used in fluorescence lifetime studies to fit the decay curves effectively. The general form of the tri-exponential decay equation is as follows:

$$f\left(t\right)=A_{1}\exp\left(-t/\tau_{1}\right)+A_{2}\exp\left(-t/\tau_{2}\right)+A_{3}\exp\left(-t/\tau_{3}\right)$$

where A1, A2, and A3 are the coefficients of the three exponential functions, representing the relative intensities of different decay components; τ1, τ2, and τ3 are the time constants of the three exponential functions, indicating the speeds of different decay components. By fitting the TRPL data with the tri-exponential decay equation, three fluorescence lifetime components ( $\tau_{1}$ ; ${\;\tau }_{2},\tau_{3}$ ) and their corresponding relative intensities (A_1_, A_2_, A_3_) can be obtained. The average fluorescence lifetime can be calculated using the following formula:

$$\tau_{av}=\frac{A_{1}\times \tau_{1}^{2}+A_{2}\times \tau_{2}^{2}+A_{3}\times \tau_{3}^{2}}{A_{1}\times \tau_{1}+A_{2}\times {\;\tau }_{2}+A_{3}\times \tau_{3}}$$

**FIGURE 7:**
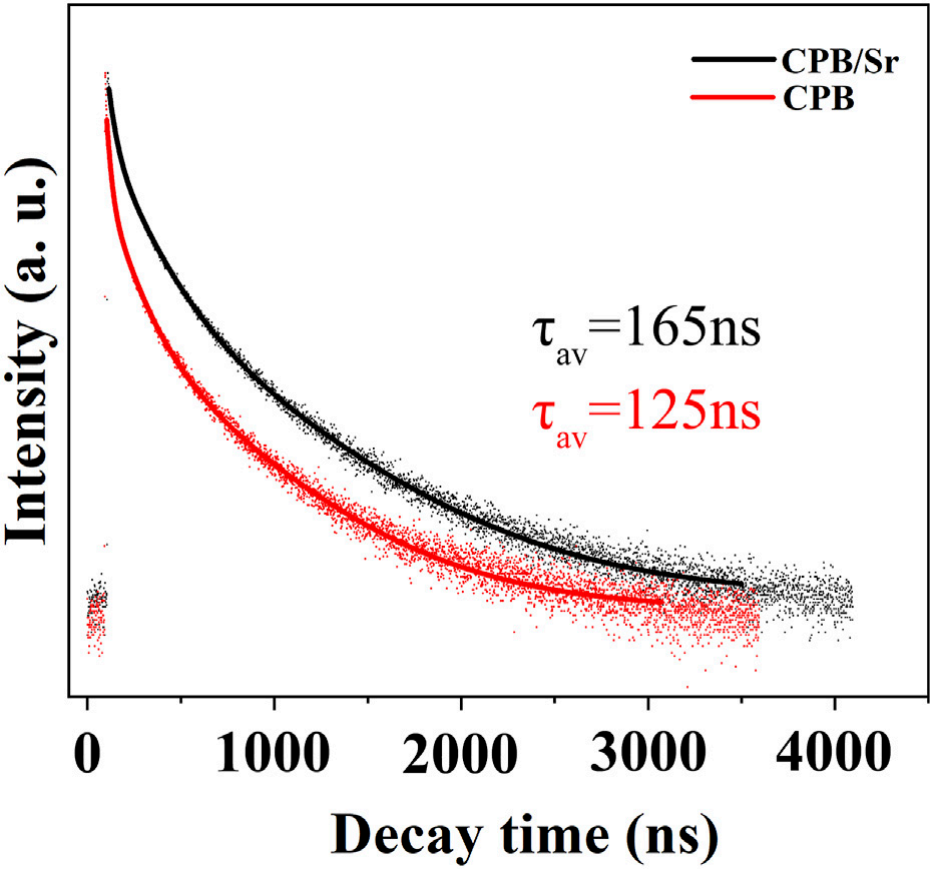
TRPL decay spectrum at 520 nm under the excitation of 375 nm for CPB/Sr and CPB.

As shown in Figure 7, the lifetime of the perovskite CPB/Sr after Sr doping is 165 ns, which is longer than the fluorescence lifetime of CPB. This result is attributed to the presence of more defects and impurity states in the undoped CPB perovskite, which can lead to non-radiative recombination of electrons and holes. Doping with Sr can partially passivate the defects on the surface of the CPB perovskite, reducing the capture and recombination of photogenerated carriers by surface states, enhancing radiative recombination, and thus prolonging the fluorescence lifetime.

## 4 Conclusion

In this study, we successfully synthesized high-quality, uniformly sized CPB/Sr perovskite nanocrystals using a low-temperature aqueous synthesis method. This approach is straightforward, easy to operate, requires minimal experimental conditions and environmental controls, and achieves a high synthesis yield. Furthermore, through microscopic characterization and optical performance analysis, our results demonstrate that the synthesized CPB/Sr exhibits remarkable stability and photoluminescent properties in aqueous environments. Specifically, the structure remains stable and the photoluminescent characteristics are largely unchanged over the first 48 h, with excellent photoluminescent properties persisting even after 264 h. Additional, TRPL confirming improved surface defect passivation, enhanced radiative recombination, and extended fluorescence lifetime. These findings further confirm that the CPB/Sr synthesized using our method demonstrates good stability and improved fluorescence performance in aqueous environments.

## Data Availability

The original contributions presented in the study are included in the article/supplementary material, further inquiries can be directed to the corresponding authors.
